# Including microbiome information in a multi-trait genomic evaluation: a case study on longitudinal growth performance in beef cattle

**DOI:** 10.1186/s12711-024-00887-6

**Published:** 2024-03-15

**Authors:** Marina Martínez-Álvaro, Jennifer Mattock, Óscar González-Recio, Alejandro Saborío-Montero, Ziqing Weng, Joana Lima, Carol-Anne Duthie, Richard Dewhurst, Matthew A. Cleveland, Mick Watson, Rainer Roehe

**Affiliations:** 1 Institute of Animal Science and Technology, Universitat Politècnica de Valéncia, 46022 Valencia, Spain; 2 Scotland’s Rural College, Easter Bush, Edinburgh, EH25 9RG UK; 3 Instituto Nacional de Investigaciones Agrarias, 28040 Madrid, Spain; 4 Escuela de Zootecnia y Centro de Investigación en Nutrición Animal, Universidad de Costa Rica, San José, 11501 Costa Rica; 5 Genus plc, DeForest, WI 53532 USA

## Abstract

**Background:**

Growth rate is an important component of feed conversion efficiency in cattle and varies across the different stages of the finishing period. The metabolic effect of the rumen microbiome is essential for cattle growth, and investigating the genomic and microbial factors that underlie this temporal variation can help maximize feed conversion efficiency at each growth stage.

**Results:**

By analysing longitudinal body weights during the finishing period and genomic and metagenomic data from 359 beef cattle, our study demonstrates that the influence of the host genome on the functional rumen microbiome contributes to the temporal variation in average daily gain (ADG) in different months (ADG_1_, ADG_2_, ADG_3_, ADG_4_). Five hundred and thirty-three additive log-ratio transformed microbial genes (*alr*-MG) had non-zero genomic correlations (r_g_) with at least one ADG-trait (ranging from |0.21| to |0.42|). Only a few *alr*-MG correlated with more than one ADG-trait, which suggests that a differential host-microbiome determinism underlies ADG at different stages. These *alr*-MG were involved in ribosomal biosynthesis, energy processes, sulphur and aminoacid metabolism and transport, or lipopolysaccharide signalling, among others. We selected two alternative subsets of 32 *alr*-MG that had a non-uniform or a uniform r_g_ sign with all the ADG-traits, regardless of the r_g_ magnitude, and used them to develop a microbiome-driven breeding strategy based on *alr*-MG only, or combined with ADG-traits, which was aimed at shaping the rumen microbiome towards increased ADG at all finishing stages. Combining *alr*-MG information with ADG records increased prediction accuracy of genomic estimated breeding values (GEBV) by 11 to 22% relative to the direct breeding strategy (using ADG-traits only), whereas using microbiome information, only, achieved lower accuracies (from 7 to 41%). Predicted selection responses varied consistently with accuracies. Restricting *alr*-MG based on their r_g_ sign (uniform subset) did not yield a gain in the predicted response compared to the non-uniform subset, which is explained by the absence of *alr*-MG showing non-zero r_g_ at least with more than one of the ADG-traits.

**Conclusions:**

Our work sheds light on the role of the microbial metabolism in the growth trajectory of beef cattle at the genomic level and provides insights into the potential benefits of using microbiome information in future genomic breeding programs to accurately estimate GEBV and increase ADG at each finishing stage in beef cattle.

**Supplementary Information:**

The online version contains supplementary material available at 10.1186/s12711-024-00887-6.

## Background

Feed is the largest cost associated with commercial beef producers (comprising 60–70% of the variable production costs [1]), therefore any effort to achieve the most efficient ratio between feed intake and growth rate will significantly contribute to the profitability of the industry. In addition, more efficient animals are needed to address the environmental challenge faced by beef production [2, 3]. Faster growth is associated with more efficient animals [4] because they reach slaughter weight at earlier ages, saving total energy (feed) requirements for body maintenance and thus reducing impact on the environment per kg product. However, the pattern of growth rate is not stable during the finishing phase. Specifically, growth rate declines and fat deposition increases with age [5–7], which has a direct impact on their feed conversion efficiency. Understanding the biology and, for breeding purposes, the genetics underlying growth rate and its pattern along the growing life of the animal [8], and adjusting feeding requirements accordingly, are critical to maximizing the feed conversion efficiency during each of the growing phases [9]. To identify these phenotypic and genetic factors, in this study, we treated average daily gains at different growth stages as different traits [10], and analysed them using multivariate models.

Among different factors that affect bovine growth performance, the microbial metabolism in the rumen plays a key role [11, 12], since cattle obtain ~ 70% of their energy requirements from volatile fatty acids produced by microbial fermentation [13], and 50–80% of their amino acids requirements from microbial proteins [14, 15]. In addition, the ruminal microbiome produces many microbiome-derived metabolites that act as regulatory signals in the gut-brain [16] and gut-liver axis [17–19], which may influence body composition [20], bone density [21], muscle development [22] and feeding behavior [23]. Given the importance of the rumen microbiome on growth, it is likely that variations in microbial pathways are associated with fluctuations in the growth rate of beef cattle. Host genetics partially determines the rumen microbiome [24–28], as first suggested by Weimer et al*.* [29] based on an analysis of a ruminal microbiome exchange in dairy cattle. Later, several studies reported moderate heritability estimates for some microbial genera and gene abundances in several beef populations [30]. The main hypothesis of this study was that relevant genomic correlations exist between performance and microbial traits, and thus our objective was to provide evidence for a shared genetic determination of cattle growth trajectory and the functional microbiome. If these genomic correlations are informative enough, it would be possible to estimate the animal’s genetic value for growth rate with greater accuracy when combining the animal weight with abundance information of microbial genes in its rumen, or, if weight records are not available, to evaluate animals based only on their microbiome composition. Furthermore, the large amount of genomic variation on microbial abundances might open the opportunity of exploring the microbiome as a breeding tool to obtain simultaneous desired responses to selection on different traits (e.g. growth rates at different stages) by enhancing specific microbial mechanisms that have favourable genomic correlations with all of these traits.

This study used a unique database of 359 animals with weekly body weight records during the finishing period and a comprehensive identification of their functional core microbiome profiles (i.e. abundances of 3632 microbial genes (MG) present in at least 70% of the animals) from rumen samples collected at slaughter. The first objective was to investigate the genomic correlations between the rumen microbiome and the average daily gains of cattle at different stages of the finishing period. The second objective was to investigate whether genomic evaluation for average daily gain traits can be performed using indirect information from MG (i.e. microbiome-driven breeding strategy), and to evaluate the improvement in the accuracy of EBV when adding microbiome information into the genomic evaluations. The third objective was to explore whether using rumen microbiome abundances, which are genomically correlated to average daily gain at different stages in the same direction, will present any advantage on the responses in these traits.

## Methods

### Animals

Data were obtained from 359 beef cattle used in different experiments [31–35] conducted over five years (2011, 2012, 2013, 2014 and 2017). The animals were from different breeds (rotational crosses of Aberdeen Angus and Limousin breeds, Charolais crosses and purebred Luing) and were fed two basal diets consisting of 480:520 and 80:920 forage:concentrate ratios. Additional file 1: Table S1 shows the distribution of animals and data among experiments, breeds, and diets.

### Collection of weight data and estimation of average daily gains at different stages during the finishing period

**Fig. 1:**
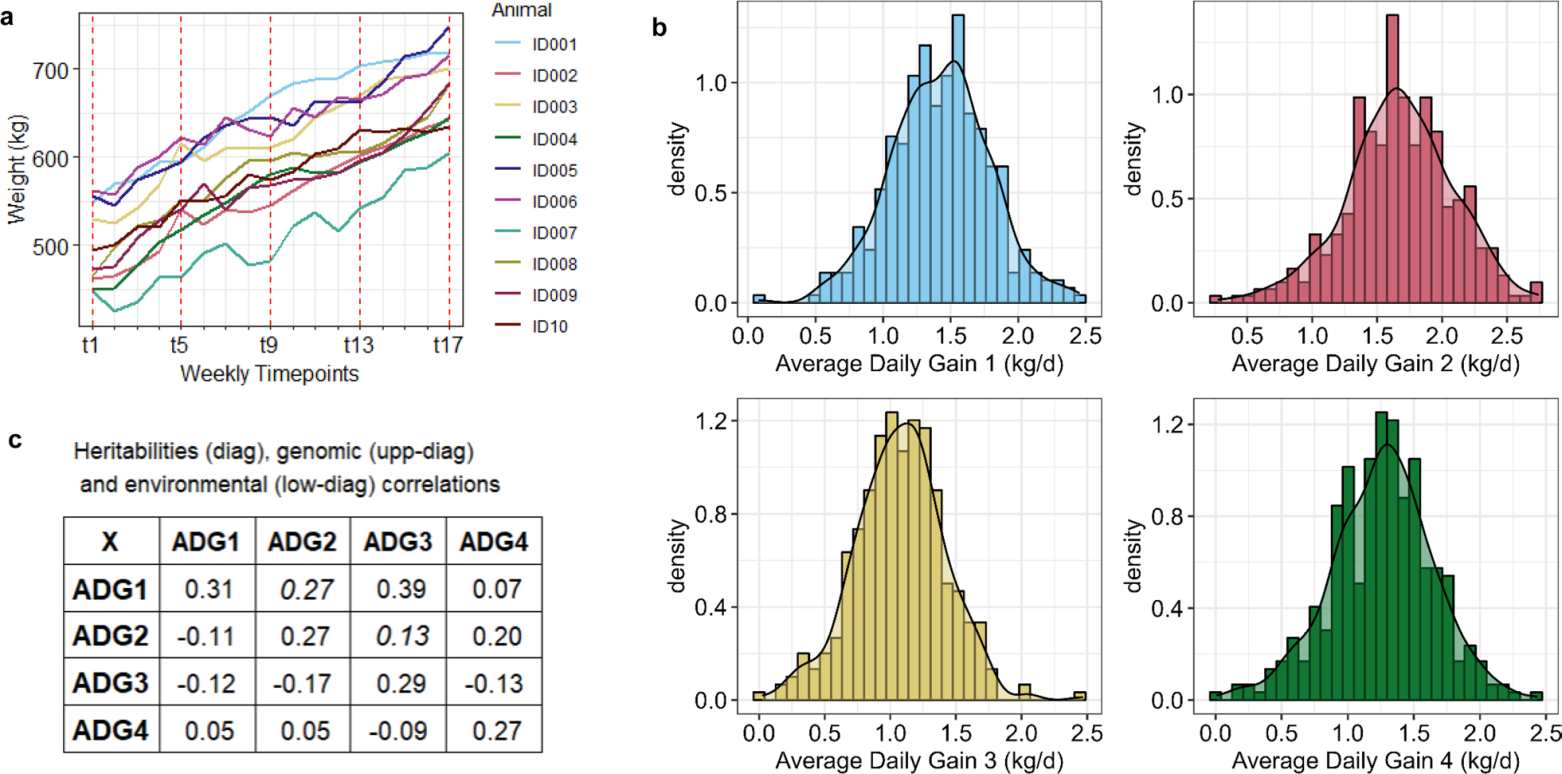
Longitudinal growth rates (ADG) at different 4-week stages during the finishing period. **a** Variation in the growth curves from 10 randomly selected animals in the study. **b** Phenotypic distribution (after correction for systematic effects) of ADG_1_, ADG_2_, ADG_3_ and ADG_4_, with decreasing means (1.57, 1.50, 1.48 and 1.41 kg/day, respectively). **c** Heritabilities, genomic and environmental correlations between ADG_1_, ADG_2_, ADG_3_ and ADG_4_

Seventeen body weights of each of the 359 beef cattle were recorded weekly for four months when the animals were between 394 ± 32 and 505 ± 33 days of age, always before feeding using a calibrated weight platform. We divided the stages of growth into four longitudinal average daily gains (ADG_1_, ADG_2_, ADG_3_ and ADG_4_), representing four consecutive 4-week intervals (Fig. 1a). Each monthly average daily gain was estimated by linear regression using five weight points, as the slope between weight increase and number of past days.

### Collection and sequencing of genomic samples

For host DNA analysis, 6–10 mL of blood from the 359 beef cattle were collected from the jugular or coccygeal vein on live animals or during slaughter in a commercial abattoir. An additional seven blood and 23 semen samples from sires of the cattle were available. Blood was stored in tubes containing 1.8 mg EDTA/mL blood and immediately frozen to – 20 ºC. Genomic DNA was isolated from blood samples using the Qiagen QIAamp toolkit and from semen samples using the Qiagen QIAamp DNA Mini Kit, according to the manufacturer’s instructions. The DNA concentration and integrity were estimated using a Nanodrop ND-1000 (NanoDrop Technologies). Genotyping was performed by Neogen Genomics (Ayr, Scotland, UK) using the GeneSeek Genomic Profiler (GGP) Bovine single nuleotide polymorphism (SNP) 50k Chip (GeneSeek, Lincoln, NE). Missing SNP genotypes were imputed using the Beagle 5.2 software [36]. Genotypes were filtered for quality control purposes using PLINK version 1.09b [37]. SNPs were removed from further analysis if they met any of these criteria: unknown chromosomal location according to Illumina’s maps [38], SNP call rate lower than 95%, deviation from Hardy–Weinberg proportions (χ^2^ test *P-value* > 10^–8^), or minor allele frequency lower than 0.05. Animals showing genotypes with a call rate lower than 90% were also removed. After imputation and filtering, 386 animals and 38,807 SNPs remained for the analyses.

### Collection and sequencing of metagenomic samples and compositional transformation of microbial abundances

For microbial DNA analysis, *post-mortem* digesta samples (approximately 50 mL) were collected from 359 cattle at slaughter, immediately after the rumen was opened to be emptied. All animals were moved from their pens and restricted to feed access between 4 to 5 h prior to slaughter. Five mL of the strained rumen fluid were mixed with 10 mL of phosphate buffered saline (PBS) with glycerol (87%) and stored at − 20 °C. DNA extraction from rumen samples was performed according to the protocol of Yu and Morrison [39] based on repeated bead beating with column filtration, and DNA concentrations and integrity were assessed using the same procedure (Nanodrop ND-1000) as for blood samples. DNA Illumina TruSeq libraries were prepared from microbial genomic DNA and sequenced on an Illumina HiSeq Systems 4000 (samples from 283 animals from experimental years 2011, 2012, 2013, and 2014) [40, 41] or a NovaSeq (samples from 76 animals from experimental year 2017) by Edinburgh Genomics (Edinburgh, Scotland, UK). Paired-end reads (2 × 150 bp for HiSeq systems 400 and NovaSeq) were generated, ranging from 7.8 to 47.8 GB per sample (between 26 and 159 million paired-end reads). To measure the abundance of known functional MG, the Kyoto Encyclopedia of Genes and Genomes (KEGG) Orthologue (KO) abundance pipeline KOunt [42] was used (https://github.com/WatsonLab/KOunt). Whole metagenome sequencing reads were trimmed for quality using the Fastp tool [43] and assembled using the MEGAHIT assembler [44]. Proteins were predicted using the Prodigal software [45], filtered to remove incomplete proteins, and searched against the KEGG database (https://www.genome.jp/kegg/ko.html) [46] (version 2020-10-04) using the KofamScan tool [47]. Hits that exceeded the default KofamScan thresholds were assigned to KO. Proteins that exceeded the threshold for multiple KO or that had no hits were grouped separately. The resulting KO grouping corresponded to a very similar set of sequences. The BWA-MEM algorithm [48] was used to map reads against their assembly and KO abundance was calculated using the BamDeal [49] and BEDTools tools [50]. We identified 7976 KO, which are referred to as MG. To discard non-core microbiome functions, we used only MG that were present in at least 252 of the 359 (70%) samples which resulted in 3632 MG representing 99.55% of the total counts identified in the microbiome dataset. Remaining zeroes were imputed based on a geometrical Bayesian-multiplicative method [51]. To deal with data compositionality, MG abundances were additive log-ratio transformed (*alr*) using the *ribulose-phosphate 3-epimerase* gene (*rpe*; EC:5.1.3.1; KEGG code K01783) as a reference, resulting in 3631 additive log-ratio transformed microbial gene abundances (*alr*-MG). The criteria to select the reference MG was a trade-off between two conditions explained in Greenacre et al. [52]: first, a Procrustes correlation of 0.9974 between the exact log-ratio geometry and the approximate geometry generated by the set of *alr*-MG, which ensures that Euclidean distances between samples are preserved after the transformation and second, a low variance of 0.0379 in the log relative abundance of *rpe* (coefficient of variation of 5.08%), which further facilitates the *alr* interpretation by reducing it to the numerator part.

The main approach of this study was to explore the genomic associations between longitudinal growth rate and microbiome function, defined as *alr*-MG. Also of interest was the main microbial taxa in our populations associated with the *alr*-MG. To this end, we identified which of the 4941 rumen uncultured genomes (RUG) generated by Stewart et al. [40] had been annotated with our *alr*-MG of interest.

### Estimation of the heritability (h^2^) of the functional core microbiome and its genomic correlations (r_g_) with longitudinal average daily gains

Heritabilities of the functional core microbiome were estimated by fitting the 3361 *alr*-MG as observed traits in 3361 univariate genomic models. Each model was as follows:

1 $$\mathbf{y}=\mathbf{X}\mathbf{b}+\mathbf{Z}\mathbf{u}+\mathbf{e}.$$

Phenotypic data were assumed to be conditionally normally distributed as:

2 $$\mathbf{y}|\mathbf{b},\mathbf{u},{\upsigma }_{{\text{e}}}^{2}\sim {\text{N}}\left(\mathbf{X}\mathbf{b}+\mathbf{Z}\mathbf{u},{\mathbf{I}\upsigma }_{{\text{e}}}^{2}\right),\boldsymbol{ }$$

where $\mathbf{y}$ is the vector containing the observed *alr*-MG for each of the 359 animals, $\mathbf{b}$ is the vector including a combination of diet, breed and experimental year (17 levels) as fixed effects, $\mathbf{u}$ is the vector of host genomic random effects, $\mathbf{e}$ is the vector of residuals, and $\mathbf{X}$ and $\mathbf{Z}$ are the known incidence matrices relating fixed and host genomic effects, respectively, to the microbial abundance observations. Note, that sequencer effect is nested within year and thus accounted for by including this effect in the model. Random effects had a normal distribution with mean 0 and variances ${\mathbf{G}}_{\mathbf{G}\mathbf{R}\mathbf{M}}{\upsigma }_{{\text{u}}}^{2}$ for $\mathbf{u}$ and ${\mathbf{I}\upsigma }_{{\text{e}}}^{2}$ for $\mathbf{e}$, where ${\upsigma }_{{\text{u}}}^{2}$ and ${\upsigma }_{{\text{e}}}^{2}$ are the genomic and residual variances, ${\mathbf{G}}_{\mathbf{G}\mathbf{R}\mathbf{M}}$ is the genomic relationship matrix computed following Method 2 of Van Raden [53] and $\mathbf{I}$ is an identity matrix of the same order as the number of individuals with data. Bayesian statistics were used [54] to obtain and interpret the results. The analyses were carried out using the BGLR software [55]. The fixed effects were assigned flat priors (i.e., Gaussian prior with a null mean and a very large variance). The random variances were assigned default priors; i.e. scaled-inverse chi square distributions with the prior degree of freedom (df0) equal to 5 and the prior scale parameter S0 = var(Y)*(df0 + number of traits + 1)*R^2^, var (Y) being the phenotypic variance of the trait and R^2^ being defined as the proportion of variance that one expects, a priori, to be explained by the regression with 0.5 as default value [56]. As features of the marginal posterior distributions of h^2^, we calculated the median and their highest posterior density interval at 95% probability (HPD_95%_). To test the sensitivity of our estimates from different prior information, we randomly selected 100 *alr*-MG and computed their h^2^, this time using scaled-inverse chi square distributions with different df0 (1, 10 and 20) for genomic variances. We also investigated the distribution of the h^2^ for the *alr*-MG under a null hypothesis of no connection between *alr*-MG phenotypes and genotypes; i.e., we re-computed the h^2^ of the 3631 *alr*-MG after permuting the data (1000 repetitions) and compared their distribution to the real distribution of h^2^.

The genomic correlations between the 3631 *alr*-MG and each of the average daily gains (ADG_1_, ADG_2_, ADG_3_ and ADG_4_) were estimated by fitting 4 times 3631 bivariate animal models with the same effects as for Model (1). In this case, the phenotypic data were assumed to be conditionally normally distributed as:

3 $$\mathbf{y}|\mathbf{b},\mathbf{u},\mathbf{R}\sim {\text{N}}\left(\mathbf{X}\mathbf{b}+\mathbf{Z}\mathbf{u},\mathbf{R}\right),$$

where $\mathbf{y}$ is a vector containing the observed average daily gain and *alr*-MG for each of the 359 animals, $\mathbf{b}$, $\mathbf{u}$, $\mathbf{X}$ and $\mathbf{Z}$ are as previously described but extended to a bivariate approach. Random effects had a multivariate normal distribution with a mean 0 and variances $\mathbf{G}\otimes {\mathbf{G}}_{\mathbf{G}\mathbf{R}\mathbf{M}}$ for $\mathbf{u}$ and $\mathbf{R}\otimes \mathbf{I}$ for residuals, where $\mathbf{G}$ and $\mathbf{R}$ are the 2 × 2 genomic and residual (co)variance matrices, respectively, and $\mathbf{I}$ is an identity matrix of the same order as the number of individuals with data. Models were solved using the BGLR software. As before, the fixed effects were assigned flat priors. The (co)variances for the residual and the genomic effects were assigned priors with default hyper parameters set in the unstructured option offered by the software. This is, an inverse Wishart distribution with five and three prior df0 for the residual and the genomic effects, and a prior scale 2 × 2 matrix equal to **S0** = var(Y)*(df0 + 1 + number of traits)* R^2^, **var(Y)** being the 2 × 2 phenotypic variance–covariance matrix of the traits and a default value [56] of 0.5 for R^2^. As features of the marginal posterior distributions of r_g_, we calculated the median and their HPD_95%_. We also computed the probability of the r_g_ being higher or lower than 0 when the median is positive or negative, respectively (P_0_). Non-zero genomic correlations were defined as the estimates with P_0_ ≥ 0.85. To test the sensitivity of our estimates from different prior information, we re-computed these r_g_, this time, using inverse Wishart distributions with different df0 (2, 10 and 20) for genomic effects.

In both the univariate and bivariate analyses, the marginal posterior distributions of the estimated parameters were based on Markov Monte Carlo chains consisting of 500,000 iterations, with a burn-in period of 100,000, and only one of every 50 samples was saved for inferences. Convergence was tested with the R package *coda* [57] by checking the Z criterion of Geweke. Monte Carlo sampling errors were computed using time-series procedures and checked for being at least 10 times lower than the standard deviation of the posterior marginal distribution [54].

### Heritabilities and r_g_ between longitudinal average daily gains at different stages of the finishing period

To estimate the h^2^ of ADG_1_, ADG_2_, ADG_3_ or ADG_4_ and their r_g_, we fitted six bivariate genomic models including pair-wise combinations of the four longitudinal growth traits as observations. The bivariate models included the same effects as described for Model (1). Phenotypic data was assumed to be conditionally normally distributed as described for Model (3). The analyses were carried out using the BGLR software [55], with the same priors as the default ones defined for Model (3). The marginal posterior distributions of the h^2^ were computed averaging the three h^2^ marginal posterior distributions obtained from three bivariate models involving each average daily gain trait. To describe the h^2^ and r_g_ estimates, we calculated the median, HPD_95%_ and P_0_ (only for r_g_) of their final marginal posterior distributions.

### Selection of microbiome variables to maximize accuracy of estimated genomic breeding values of longitudinal average daily gains

For breeding purposes, we considered only the *alr*-MG that had a genetic correlation r_g_ with any of the average daily gains achieving P_0_ ≥ 0.85. From those, we discarded the *alr*-MG for which the numerators were MG with average relative abundances across all animals ≥ 0.001%. The microbial gene *rpe* used as denominator had a mean relative abundance of 0.03%. We filtered for a minimum abundance of 0.001% since microbial genes that show low counts in the population are more likely to be unidentified in the rumen of some animals and these generated counts may be subject to more technical variation [58, 59]. The microbiome-driven breeding strategy was based on fitting a multi-trait best linear unbiased prediction (BLUP) model, which includes the goal traits and the selected *alr*-MG as observed traits [60]. Because the number of coefficients to be estimated and the computational cost increase rapidly with the number of traits included in the model, our aim was to select a reduced subset of 32 *alr*-MG among all the candidates, which maximize the accuracy of the EBV of the average daily gain traits. We selected two alternative subsets of 32 *alr*-MG, referred to as non-uniform and uniform r_g_ subsets. The first subset (non-uniform r_g_) was selected based on a stepwise Akaike information criteria (AIC) forward regression method using the R package MASS [61], where the genomic estimated breeding values (GEBV) of average daily gain traits were the dependent variables, and the GEBV of the *alr*-MG were tested as predictors. Genomic EBV for all traits were obtained from the corresponding univariate analysis described above. First, the best *alr*-MG to predict ADG_1_ was selected based on the largest AIC reduction, in a model already including the remaining average daily gain traits ADG_2_, ADG_3_ and ADG_4_ as predictors. Next, the best *alr*-MG to predict ADG_2_ was added to the model based on the same criterion, in a model already including the remaining ADG traits (ADG_1_, ADG_3_ and ADG_4_) plus the previously selected *alr*-MG. The one-by-one addition of variables to the model continued for ADG_3_ and ADG_4_, and then started again for ADG_1._ We stopped the process after selecting 32 *alr*-MG in the final model (8 per average daily gain trait). The second subset (uniform r_g_) was selected by the same procedure, but restricting the selection of *alr*-MG to those that had an r_g_ with ADG_1_, ADG_2_, ADG_3_ and ADG_4_ with the same sign, regardless of their magnitude. The reason for this strategy was to test whether the exclusive use of microbial functions with favourable associations with overall growth traits would provide an advantage in terms of the accuracy of the GEBV of ADG.

### Multitrait BLUP model to evaluate the expected benefits of including *alr*-MG in the genomic evaluation of average daily gain traits

The GEBV of ADG_1_, ADG_2_, ADG_3_ and ADG_4_ were estimated based on three strategies:

1. Using only ADG_1_, ADG_2_, ADG_3_ and ADG_4_ information (direct strategy).
2. Using only information of the subset of 32 *alr*-MG (microbiome-driven breeding strategy), considering the two non-uniform and uniform r_g_ alternative subsets.
3. Using information from ADG_1_, ADG_2_, ADG_3_ and ADG_4_ and the subset of 32 *alr*-MG (combined strategy), considering the two non-uniform and uniform r_g_ alternative subsets.

In strategy (1), GEBV of average daily gains were estimated by fitting a tetra-variate genomic (G)BLUP analysis that included observed ADG_1_, ADG_2_, ADG_3_, and ADG_4_ traits, providing the previously computed coefficients of the 4 × 4 genomic and residual (co)variance matrices as fixed. In strategies (2) and (3), GEBV of average daily gains were estimated by fitting a 36-trait multivariate analysis, including the 32 *alr*-MG and missing [strategy (2)] or observed values [strategy (3)] for average daily gain traits, and introducing the coefficients of the corresponding 36 × 36 genomic and residual (co)variance matrices as fixed. In a previous step, each coefficient of these matrices was estimated by fitting a bivariate model (496 models in total) with the corresponding pairwise combination between *alr*-MG as observed traits. Models were identical to those used to compute genomic parameters between *alr*-MG and average daily gain traits. Once the 36 × 36 residual and genomic variance–covariance matrices were obtained, the latter needed bending in order to be positive definite (tolerance for minimum eigenvalues = 1 × 10^–3^). The difference between original and bent matrices was never larger than the posterior standard error of the corresponding parameters, and none of the h^2^ of the trait after bending varied by more than 5% from their original values.

The GEBV estimates were based on Monte Carlo Markov chains, which consisted of 100,000 iterations with a burn-in period of 20,000. To reduce autocorrelation, only 1 of every 100 samples was saved for inference. The GEBV accuracies of ADG_1_, ADG_2_, ADG_3_ and ADG_4_ under each strategy were computed as follows:

4 $${{\text{Accuracy}}}_{{\text{i}}}=\sqrt{1-\frac{{{\text{sd}}}_{{\text{i}}}^{2}}{{{{\text{g}}}_{{\text{RM}}}}_{{\text{ii}}}\mathbf{*}{\upsigma }_{{\text{u}}}^{2}}},$$

where $\text{sd}_\text{i}$ is the standard deviation of the posterior marginal distribution of the GEBV for animal $\text{i}$, ${{{\text{g}}}_{{\text{RM}}}}_{{\text{ii}}}$ is the diagonal element of the genomic relationship matrix for animal $\text{i}$ and ${\upsigma }_{{\text{u}}}^{2}$ is the genomic variance of the trait. For the three strategies, prediction of the selection response in ADG_1_, ADG_2_, ADG_3_ and ADG_4_ was estimated by ranking individuals based on their GEBV for each of the four traits [62], assuming equal economic weights for all the traits, and a selection intensity of 1.755 or proportion of selection of 10%. Prediction of the response to selection for each trait was estimated as the marginal posterior distribution of the difference between the mean estimated GEBV of all animals and the mean of the selected animals, and its median and standard deviation were calculated.

## Results

### Genomic determination of average daily gains at different stages during the finishing period

Average daily gain was largest in the first month (ADG_1_ average was 1.57 kg/day), and then decreased slightly over time (ADG_2,_ ADG_3_ and ADG_4_ averaged 1.50, 1.48 and 1.41 kg/day), with all traits showing a similar adjusted phenotypic variation across animals (coefficient of variation ranging from 25 to 32%, after correction for diet, breed, and year effects). Around 30% of this phenotypic variation was due to genomic variation across animals in all growth stages, as the h^2^ of ADG_1_, ADG_2_, ADG_3_ and ADG_4_ were 0.31 (HPD_95%_ of [0.13, 0.48]), 0.27 [0.12, 0.42], 0.29 [0.13, 0.45] and 0.27 [0.11, 0.43], respectively. In general, a common positive genomic association underlaid the average daily gain over the four considered months of the finishing period, although the magnitude of these genomic correlations (r_g_) were moderate or low and ranged from 0.39 [0.03, 0.69] between ADG_1_ and ADG_3_ (with a probability of r_g_ being different from 0 (P_0_) = 0.97) to 0.07 [− 0.34, 0.47] between ADG_1_ and ADG_4_ (P_0_ = 0.63) (see Fig. 1c). An exception was the r_g_ between ADG_4_ and ADG_3_, which was negative with a probability P_0_ of 0.72, but the estimate had a low median of -0.13 and large HPD_95%_ of [− 0.50, 0.28] due to the small size of the dataset. These results suggest that different host genes influence the growth rate at different growth stages [10]. The environmental correlations between longitudinal traits were low (ranging from − 0.17 to 0.05) and inconsistent with r_g_, indicating that the random environmental factors were not correlated during the growth of cattle.

### Heritabilities of functional core microbiome and r_g_ between average daily gains at different stages and the functional core microbiome

The functional composition of the rumen microbiome was influenced by the host genetics (see Additional file 2: Table S2). The h^2^ estimates of the 3631 *alr*-MG ranged from 0.19 [0.10, 0.29] to 0.44 [0.22, 0.68]. However, the estimated variance components were not entirely independent from the prior information used in the Bayesian analyses (see Additional file 2: Table S2, Additional file 3: Figure S1 and Additional file 4: Figure S2) due to the limited size of the dataset. Next, we investigated whether the 3361 *alr*-MG were genomically correlated with any of the longitudinal average daily gains (ADG_1_, ADG_2_, ADG_3_ and ADG_4_) and therefore could be used to indirectly estimate their GEBV. Of the 3631*alr*-MG, 533 had non-zero (P_0_ ≥ 0.85) r_g_ with at least one of the longitudinal traits. When the overlapping MG were also counted, there were 583 non-zero r_g_ estimates: 236 with ADG_1_, 227 with ADG_2_, 44 with ADG_3_, and 76 with ADG_4_, with median values ranging from |0.21| to |0.42| (see Additional file 5: Table S3). The magnitudes of the r_g_ estimates (medians) were not completely insensitive to the use of different prior information, particularly when using highly informative priors (see Additional file 5: Table S3 and Additional file 6: Figure S3).

Among the 533 *alr*-MG with non-zero r_g_ (P_0_ ≥ 0.85) with any of the average daily gains, the largest overlap occurred between ADG_1_ and ADG_3_ (n = 8) followed by that between ADG_1_ and ADG_2_, (n = 32). These results suggest that part of the host genetic determinism that underlies average daily gains at different stages occurs through differences in host genes that influence the functional core microbiome in the rumen. The functionalities of *alr*-MG with non-zero r_g_ with average daily gain at earlier growth stages (either with ADG_1_, ADG_2_ or both), included specific microbial energy-related processes; for example, several subunits of the *energy-converting hydrogenase A* (*ehaA*, *ehaC, ehaH, ehaK*) and *B* (*ehbC, ehbE, ehbI, ehbJ, ehbO, ehbQ, ehbP*) gene groups presented positive r_g_ (medians from 0.22 to 0.30, P_0_ ≥ 0.85), while different subunits of the *F-type H* + *transporting ATPase* (*ATPF0A*, *ATPF0B*, *ATPF0C*, *ATPF1E*, *ATPF1G*) gene group presented negative r_g_ (medians from − 0.26 to − 0.37, P_0_ ≥ 0.88). Earlier average daily gains were also correlated with a large number of *alr*-MG (n = 31) involved in ribosome biogenesis. Among these, 13 had non-zero positive r_g_ from 0.22 to 0.34 only with ADG_1_ (e.g. *NMD3*, *SDO1*, *RP-L18Ae*, and *RP-L10e*) and 13 had non-zero negative r_g_ from − 0.24 to − 0.34 only with ADG_2_ (e.g. *RP-L4*, *RP-L5*, *RP-L6*, and *RP-L7*). Moreover, average daily gains at early stages (ADG_1_ and ADG_2_) had strong and positive r_g_ with 11 *alr*-MG involved in methane metabolism including *methyl-coenzyme M* (*mcrG*), *formylmethanofuran* (*ftr, fwdD*)*, formate dehydrogenase* (*fdhB*) or *coenzyme F*_*420*_* hydrogenase* (*frhB*) (medians from 0.22 to 0.30, P_0_ ≥ 0.85).

Among the microbial mechanisms that were linked (non-zero r_g_) to ADG at later stages of the finishing period (either with ADG_3_, or ADG_4_), the metabolism of sulphur was largely represented, and included three *alr*-MG with negative r_g_ with ADG_4_ (*asrC*, *asrB* and *hydB,* all with an r_g_ median of − 0.24, P_0_ ≥ 0.86) and three *alr*-MG with positive r_g_ with ADG_3_ (*moeB*, *MOCS2B* and *dmsB*, with r_g_ medians ranging from 0.23 to 0.29, P_0_ ≥ 0.86). Interestingly, different *alr*-MG that are also involved in sulphur metabolism had non-zero positive or negative r_g_ with ADG_1_ and ADG_2_ (*thiS*, *NFU1*, *hydG*, *tusA*). Another microbial mechanism that influences growth rates at later stages was the metabolism of amino and nucleotide sugars (*galt, nanA* and *nanE* had negative r_g_ with ADG_4_ with a median of − 0.24, P_0_ ≥ 0.85), and as before, a few other *alr*-MG in the same pathway correlated positively with ADG_1_ and ADG_2_ (*legF*, *E1.1.1.374*, and *pgu* with r_g_ ranging from 0.26 to 0.34, P_0_ ≥ 0.89).

Although in some cases our results allowed us to associate specific microbial mechanisms to specific growing stages (see above), a common situation was that *alr*-MG that are involved in specific microbial metabolic pathways presented positive or negative non-zero (P_0_ ≥ 0.85) r_g_ with different longitudinal average daily gains without a clear pattern, which reflects the complexity of the host-microbiome regulation during the four months of growth analysed in this study. This was the case for 27 *alr*-MG that are involved in the biosynthesis of several amino acids (glycine, serine and threonine; lysine; arginine; cysteine and methionine, phenylalanine tyrosine and tryptophan; or alanine) and 23 *alr*-MG that are involved in the metabolism of carbohydrates (pentose and glucoronate interconversions; fructose and mannose; galactose; starch and sucrose) all with r_g_ medians ≥|0.22|. Another example were the *alr*-MG that are linked to the synthesis of microbial cell wall or membrane components. While some of them that are involved in the biosynthesis of lipopolysaccharides (*lpxK, lpxH*, *lptA*, *lptB*, *lptC*, and *ABC-2. LPSE.P*) and peptidoglycans (*spoVD* and *ftsI*) had positive or negative r_g_ with ADG_1_ and ADG_2_ (r_g_ between |0.22| and |0.36|, P_0_ ≥ 0.85); others that are linked to the biosynthesis of O-antigens (*wecE*, *wecB*, *pseB* and *pseF*) had positive or negative r_g_ to ADG_2_ and ADG_4_ (r_g_ from |0.24| to |0.29|, P_0_ ≥ 0.85).

### Rumen uncultured genomes (RUG) associated to the 533 *alr*-MG with non-zero r_g_ with longitudinal growth rates

Only nine of the 533 *alr*-MG numerators were identified in the genome of 4877 RUG out of the 4941 RUG present in the rumen of a subset of 282 cattle from our study, described by Stewart et al. [40] (see Additional file 7: Table S4). The remaining 524 MG were not found in any of the RUG. This could be attributed to the fact that a large part of the data was lost during the binning process and to the removal of low quality RUG; and also to the different methodologies to obtain the MG information [40]. The nine MG were involved in amino acid (*trpA*, *metN*, *ABC.PE.P1* and *ABC.PA.S)* and lipid (*INO1*) transport and metabolism, genetic microbial processes (*polC* and *RP-L6*) or were not characterized (*K07133*). Their corresponding *alr*-MG were genomically correlated (P_0_ ≥ 0.85) with either ADG_1_ (*trpA*, *metN*, and *ABC.PE.P1*), ADG_2_ (*RP-L6*, *K07133*, and *INO1*), ADG_3_ (*nuoJ* and *ABC.PA.S*) or ADG_4_ (*polC*). The most ubiquitous MG were *K07133* (found in the genome of 3636 out of the 4877 different RUG), *ABC.PA.S* (in 3042 RUG), *RP-L6* (in 2921 RUG), *trpA* (in 2538 RUG) and *polC* (in 2436 RUG). The KEGG *K07133* was the most represented MG within the genome of 3636 RUG; for example, 378 of these RUG carried between 10 and 64 unique proteins classified as *K07133* in their genome; most of them are identified as *uncultured Bacteroidales bacterium* (67 RUG), *uncultured Bacteroidia bacterium* (62 RUG) or *uncultured Bacteroidetes bacterium* (49 RUG).

Of the 4877 RUG, we identified 820 that carried at least six of the nine MG. Among these, 276 were classified as *uncultured Lachnospiraceae bacterium*; 92 as *Ruminococcaceae bacterium*; 61 as *uncultured Clostridiales bacterium*; 54 as *uncultured Methanobrevibacter sp*.; 40 as *uncultured Erysipelotrichaceae bacterium*; 35 as *uncultured Succiniclasticum sp*., 32 as *uncultured Ruminococcus sp*.; 31 as *Fibrobacter succinogenes* and 26 as *uncultured Prevotellaceae bacterium*.

### Selection of an optimal subset of microbial gene abundances for genomic evaluations of average daily gains at different stages

**Fig. 2:**
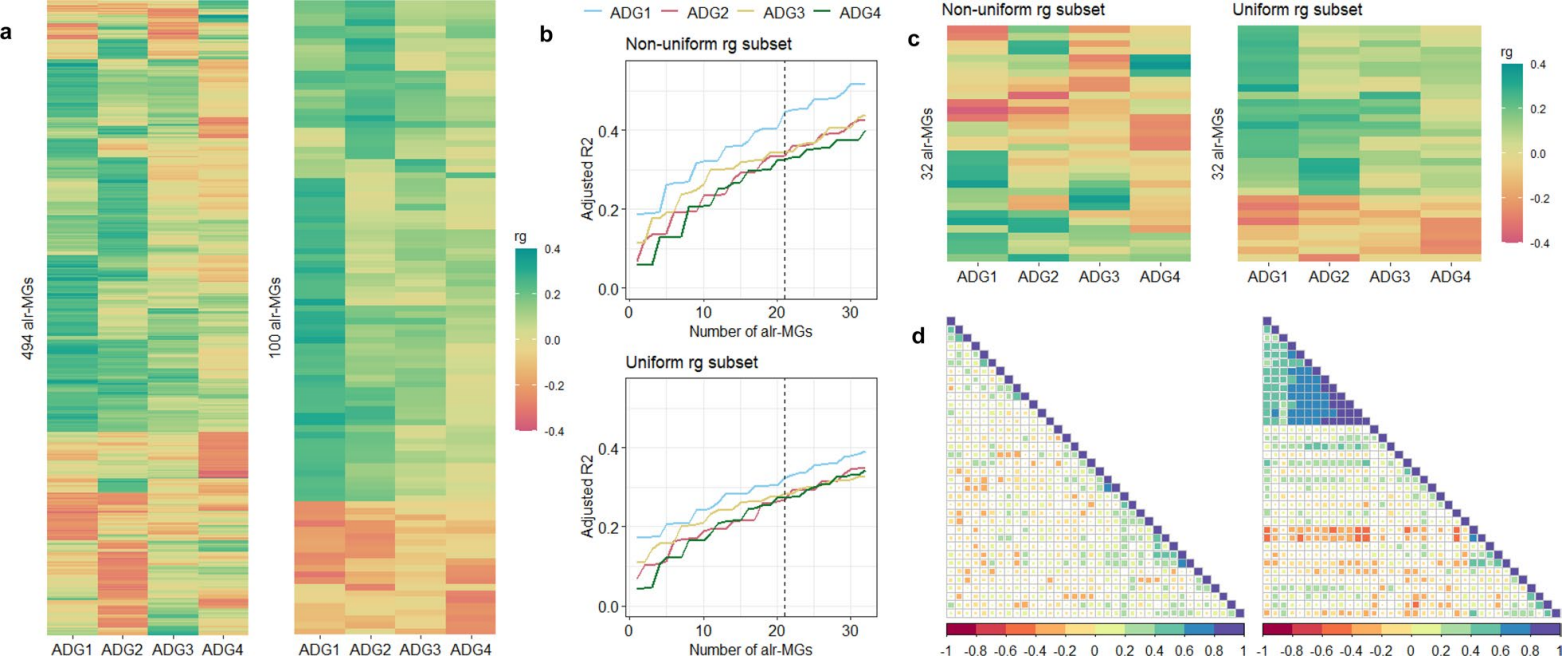
Selection of additive log-ratio transformed microbial gene (*alr*-MG) abundances to be included in genomic evaluations.** a** Host-genomic correlations (r_g_) between average daily gains at different stages of the finishing period (ADG_1_, ADG_2_, ADG_3_ and ADG_4_) with (see the left side of the panel) 494 *alr*-MG with mean relative abundance of the numerator ≥ 0.001%, and a genomic correlation (r_g_) with any of the average daily gain traits (ADG_1_, ADG_2_, ADG_3_, ADG_4_) being different from 0 with a probability ≥ 0.85; or with (see the right of the panel) the 100 *alr*-MG with the additional restriction of having a uniform r_g_ sign with all growth traits. **b** Change in adjusted R^2^ with an increasing number of *alr*-MG GEBV to 32 as explanatory variables using forward-wise linear regression to predict the GEBV of ADG_1_, ADG_2_, ADG_3_ and ADG_4_ for both alternatives defined in panel **a**. **c** Host-genomic correlations (r_g_) between average daily gains and the 32 *alr*-MG selected for both alternatives defined in panel **a**. **d** 36-traits genomic correlation matrix including the 32 *alr*-MG of the uniform (left) and non-uniform (right) r_g_ subsets and average daily gain traits

Of the 533 *alr*-MG candidates to be used for average daily gain genomic evaluations, we considered only 494 *alr*-MG with an average relative abundance across all animals ≥ 0.001% for the selection of the non-uniform r_g_ subset (Fig. 2a left). The uniform r_g_ subset was selected only from 100 of these 494 *alr*-MG that all had r_g_ estimates with all average daily gains with the same sign, regardless of their magnitude (Fig. 2a right). In both cases, the variables were chosen based on a forward-wise variable selection (see Additional file 8: Table S5). The non-uniform r_g_ subset of 32 *alr*-MG achieved greater adjusted R^2^ in the regression predicting GEBV of average daily gains (0.52, 0.42, 0.44, and 0.40 for ADG_1_, ADG_2_, ADG_3_ and ADG_4_) than the uniform r_g_ alternative (0.40, 0.35, 0.32 and 0.34, respectively, Fig. 2b). Only four *alr*-MG were common in both subsets (*ehbO*, *mshG*, *srlD*, and *traN*). The uniform r_g_ subset contained a smaller number of *alr*-MG with a non-zero r_g_ with ADG_3_ and ADG_4_ (5 with ADG_4_ and 2 with ADG_3_) than the non-uniform r_g_ alternative (7 with ADG_4_, 5 with ADG_3_ and 1 with both) (Fig. 2c). The genomic correlation matrix among the 32 *alr*-MG had a lower degree of redundancy in the first alternative, i.e. the maximum r_g_ between *alr*-MG was 0.68 (P_0_ = 0.87); while in the uniform r_g_ subset, the maximum r_g_ was 0.88 (P_0_ = 0.95) (Fig. 2d).

### Expected benefits in the accuracy of average daily gain GEBV and predicted responses to selection when microbiome traits are included in genomic evaluations

**Fig. 3:**
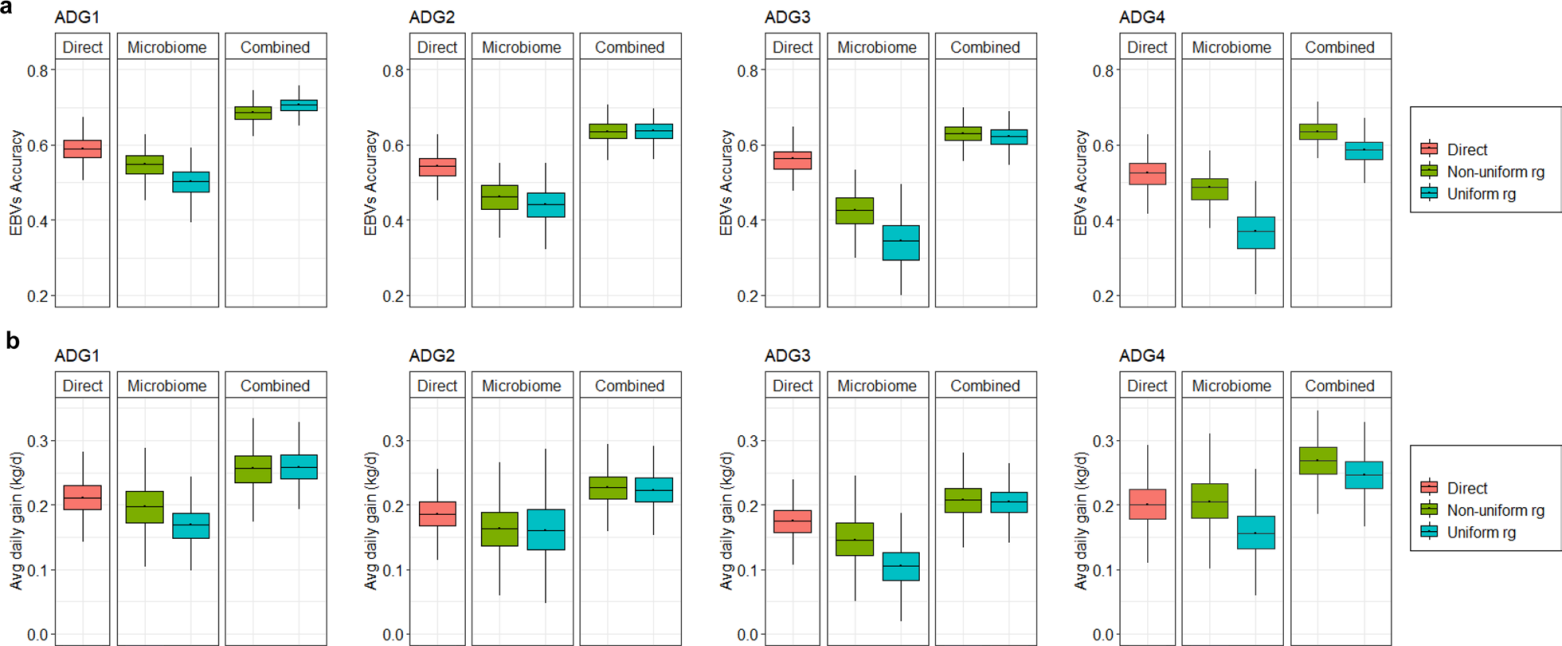
Prediction accuracies and expected predicted responses to selection on growth traits.** a** Prediction accuracy of genomic breeding values (GEBV) of growth traits (ADG_1_, ADG_2_, ADG_3_ and ADG_4_) when including exclusively each ADG trait (direct), exclusively 32 microbiome traits that were found informative for each of the ADG traits (Microbiome) or both (Combined). We used two strategies to select the 32 microbiome traits: the most informative *alr*-MG among all those that presented a non-zero genomic correlation with the ADG traits (Non-uniform r_g_ subset) or the same criterion but including the restriction their genomic correlation with all ADG traits had a uniform sign (Uniform r_g_ subset). **b** Prediction of the selection response expected for each ADG trait when selecting the 10% best animals (i.e., animals with the largest EBV for ADG_1_, ADG_2_, ADG_3_ or ADG_4_) under each strategy

We evaluated the prediction accuracy of GEBV for average daily gains at different stages as well as the prediction of selection responses in three different scenarios using different sources of information: (1) observed ADG_1_, ADG_2_, ADG_3_ and ADG_4_ (direct breeding strategy), (2) the functional microbiome information (microbiome-driven breeding strategy), and (3) the combination of observed average daily gains and functional microbiome information (combined breeding strategy). The mean GEBV accuracy in the direct breeding strategy was 0.58 ± 0.04 for ADG_1_, 0.54 ± 0.04 for ADG_2_, 0.56 ± 0.04 for ADG_3_ and 0.52 ± 0.04 for ADG_4_. Inclusion of microbiome traits in a combined approach increased the prediction accuracies of GEBV for all traits by 11 to 22% (Fig. 3a), depending on the growth trait or subset of *alr*-MG used. Using only microbiome information to estimate the GEBV of growth traits yielded reasonable accuracies ranging from 0.33 ± 0.07 to 0.55 ± 0.04 but never outperformed the direct strategy (7–41% lower accuracies relative to the direct strategy). The subset of *alr*-MG to be used as microbiome information had a stronger influence on GEBV accuracy of average daily gain when the microbiome breeding strategy was applied compared to the combined strategy. Applying the combined strategy, GEBV accuracies were similar using the non-uniform or uniform r_g_ subsets at earlier stages (0.70 ± 0.02 and 0.68 ± 0.03 for ADG_1_; 0.64 ± 0.03 and 0.63 ± 0.03 for ADG_2_; and 0.62 ± 0.03 and 0.63 ± 0.03 for ADG_3_), but using the non-uniform r_g_ subset performed better for ADG_4_ (0.58 ± 0.04 vs 0.63 ± 0.03). In the microbiome-driven strategy, the accuracies obtained were similar for both *alr*-MG subsets (0.44 ± 0.05 and 0.46 ± 0.05 for ADG_2_) but, in the non-uniform strategy, they were higher particularly at later stages (0.50 ± 0.04 vs 0.55 ± 0.04 in ADG_1_; 0.33 ± 0.07 vs 0.42 ± 0.05 in ADG_3_; and 0.36 ± 0.06 vs 0.48 ± 0.04 in ADG_4_). The lower accuracy for ADG_4_ and/or ADG_3_ using the uniform r_g_ subset is explained by the fact that most of the *alr*-MG that were penalized by the restriction were those with a high r_g_ with these two growth traits (Fig. 2a).

The magnitude of the predicted responses to selection that ranked the animals based on their GEBV for ADG_1_, ADG_2_, ADG_3_ or ADG_4_ was consistent with the GEBV prediction accuracies. Assuming a proportion of selection of 10%, the predicted responses to selection in the direct breeding strategy were 0.20 ± 0.04 kg/d for ADG_1_, 0.16 ± 0.04 kg/d for ADG_2_, 0.15 ± 0.04 kg/d for ADG_3_ and 0.21 ± 0.04 kg/d for ADG_4_ (12 to 14% of the trait mean). The expected predicted response to selection was greater when microbiome and growth traits were combined (responses ranged from 0.20 ± 0.02 to 0.27 ± 0.03 kg/d or from 14 to 19% of the trait mean), and there were no differences in terms of the two different microbial information used (0.26 ± 0.03 vs 0.26 ± 0.03 for ADG_1_; 0.23 ± 0.03 vs 0.22 ± 0.03 for ADG_2_; 0.21 ± 0.03 vs 0.20 ± 0.02 for ADG_3_; and 0.27 ± 0.03 vs 0.25 ± 0.03 for ADG_4_). However, when only microbiome information was used, a greater predicted response was obtained in later growth stages using the non-uniform compared to using the uniform r_g_ subset of *alr*-MG: 0.15 ± 0.04 vs. 0.10 ± 0.03 kg/d in ADG_3_; and 0.21 ± 0.04 vs 0.16 ± 0.04 kg/d in ADG_4_. Our results demonstrate that the inclusion of microbiome information in breeding evaluations for growth can increase the predictive accuracy of GEBV and response to selection or, if it is used alone, it is an acceptable strategy for conducting genomic evaluations for animals without growth rate records.

## Discussion

Longitudinal variations in average daily gain during cattle growth have important implications for the beef industry. For example, to achieve the highest feed conversion efficiency, nutrient requirements would have to be adjusted accordingly to the growth rate at each stage [1], which is not always practical under farming conditions. A comprehensive knowledge of the biological factors that contribute to these temporal variations offers the opportunity to modulate them towards a more uniform and increased growth pattern. One of these factors is the host’s genetic contribution, as indicated by the moderate (r_g_ = 0.31 and 0.39) to near zero (r_g_ = 0.07) or even negative (r_g_ = − 0.13) genomic correlations between early and late average daily gains during the finishing period. Several studies suggest that candidate genes that affect growth rate at different stages [63–66] are associated with skeletal muscle and adipose tissue development (e.g. *FGF4*, *PLA2G4A*, *ITGA5* or *ANGPTL4*), and some of them are also expressed in the rumen [67]. In our study, we show, for the first time, that the polygenic basis underlying average daily gains at different stages is genetically correlated with the abundance of specific functions of the rumen microbiota. This is likely to be mediated by genetically influenced traits which affect the microbial composition in the rumen, such as rumen empty weight (heavier in less efficient animals) [68], digesta retention time [69], or rumen tissue development and absorptive capacity [70–72]. The majority of the 533 *alr*-MG showed a non-zero r_g_ with average daily gain only at one of the stages, with some exceptions for ADG_1_ and ADG_2_. Regardless of the magnitude of r_g_, some of these *alr*-MG showed r_g_ with a consistent sign for earlier growing stages but changed sign as animals were older (ADG_4_), or they simply did not follow any specific pattern, contributing to the differential genetic determination underlying longitudinal growth rates. Our study relies on the assumption that the composition of the ruminal functional microbiome sampled at slaughter is reasonably representative of the composition of the rumen microbiome throughout the growth period. This is supported by the longitudinal study of Snelling et al*.* [73] that was performed with a subsample of 50 animals from our dataset. The ruminal microbiome composition was monitored over 200 days (7 time points) from the early finishing stage until slaughter, and the authors did not detect any significant variability over time, suggesting temporal stability of the core rumen microbiome [73]. In our genetic study, animals were fed two different diets (see Methods), which was included as a fixed effect in the genetic model, assuming that our strategy is applicable under these two different environments (diets). However, it is possible that there is a genetic-by-environment (GxE) interaction on growth and/or microbial traits, i.e., the genetic effects and/or microbial traits on growth can be different depending on diet. In this case, further analysis is required before the results of this study can be extrapolated to other feeding regimes. To date, the previous study of Roehe et al*.* [74] showed that there was no interaction between sire type and diet for methane emissions and that the regression coefficients between the abundance of specific microbial genes and methane emissions were similar in two groups of animals that were fed either concentrate or forage-based diets.

Growth rate is a moderately heritable low-cost indicator of feed conversion efficiency [4] because animal weigths are relatively easy to record; and therefore body weight gain is considered in almost all beef genetic selection programs [75]. Furthermore, the existence of a r_g_ (P_0_ ≥ 0.85) between specific heritable (h^2^ ≥ 0.19) microbial functions and the average daily gains reported, opens up the possibility of using microbial biomarkers as complementary information to improve the accuracy of the GEBV for growth rate (combined breeding strategy). They could even be used as the sole source of information when weights are not recorded (microbiome-driven breeding strategy), given that microbiome information can be recorded for multiple uses, such as predicting feed efficiency, methane emissions or animal health. For breeding purposes, it was necessary to overcome the high-dimensionality of the microbial metabolism that we wanted to use for selection (in our case, the 533 *alr*-MG reduced to 494 *alr*-MG after filtering for average relative abundance ≤ 0.001%), which most likely contain highly redundant information. In this study, we proposed to use a forward-wise variable selection method based on GEBV to avoid redundancy by selecting the most informative microbial predictors based on linear regression and maximizing the average daily gain explained variance. It should be noted that one limitation of this methodology is that it does not account for the prediction errors of GEBV. Recently, two alternatives approaches for using –omics data for genomic prediction have been proposed, based on a two-step application of linear mixed model equations [76] or on the extension of linear mixed models by multilayer artificial neural networks [77] that do not require selection of -omics variables. These two new approaches suggest increases on breeding value prediction accuracies similar to this study when tested on simulated data (e.g. + 12% compared to using genotypic information [76]).

A postulated advantage of a microbiome-driven breeding strategy is that the unfavourable and/or null r_g_ between productive traits (e.g., -0.13 between ADG_3_ and ADG_4_ or 0.07 between ADG_1_ and ADG_4_) may not be necessarily reflected in the overall complexity of the functional microbiome, and specific *alr*-MG with strong and sign-consistent associations to these traits could be found. The inclusion of these microbial metabolic functions in genomic evaluations could help to overcome these unfavourable correlations, but at the cost of slowing down the response on the production traits. However, we faced two difficulties when testing this hypothesis. First, due to our small dataset, we could not determine wether ADG_3_ and ADG_4_ were negatively correlated with sufficient evidence. Second, our results showed a lack of microbial functions with at least moderate r_g_ with ADG_1_ and ADG_4_ or ADG_3_ and ADG_4_. The use of *alr-*MG that are genomically correlated with average daily gain at different stages in the same direction (regardless of their magnitude) for genomic evaluations did not only provide no advantage over the non-uniform r_g_ alternative, but also reduced the accuracy of GEBV and the predicted response obtained at the latter stages. Regardless of the subset of *alr*-MG included in the genomic evaluations, a relevant finding was that combining information from *alr*-MG and average daily gains is the most beneficial scenario to increase their GEBV accuracies (the combined strategy achieved + 11–22% greater GEBV accuracy than the direct strategy), and consequently, it achieves the greatest predicted responses to selection (up to 14–19% of the mean for all growth traits). The best performance of a combined breeding strategy compared to a direct or microbiome-driven one was also observed in a study that aimed at mitigating methane emissions [60] of beef cattle. The makeup of the microbiome represents a vast pool of genomic variation which, until now, has been only indirectly exploited when targeting production traits. However, our study demonstrates that targeting the microbiome directly could bring additional progress in genomic selection programmes. One concern with our study is that the results should be taken with caution as the estimation of GEBV is very sensitive to the magnitude of the variance components used, which in our case were estimated with a database of 359 animals, and were partly influenced by the priors. Much larger databases are required to obtain more accurate estimates, but this is currently hampered by the high cost of whole metagenomic deep sequencing and the novelty of using KEGG gene abundance as selection criteria. The feasibility of selection on microbial traits was shown in a selection experiment in pigs based on the abundance of *Prevotella*, *Mitsuokella*, *Treponema* and *Ruminoccocus* in faeces, in which responses to selection in their abundances and correlated responses in growth rate and back fat thickness were obtained [78]. In our case, we proposed *alr*-MG as selection information because, according to our previous work, they are more influenced by the host genome and more informative for phenotypic as well as genomic evaluation of traits than taxonomic composition [60, 74].

The targeted microbial metabolic routes for genomic selection in our microbiome-driven or combined breeding strategy can be biologically interpreted based on the functions of the numerators of *alr*-MG, given that the denominator has a very reduced variation [52]. We found that the *alr*-MG involved in the biosynthesis of ribosomes and DNA replication (*NMD3*, *SDO1*, *RP-L18Ae*, and *RP-L10e*), energy-related processes (*ehaA*, *ehaC*, *ehaH*, *ehaK*, *ehbC*, *ehbE*, *ehbI*, *ehbJ*, *ehbO*, *ehbQ*, and *ehbP*) and methane metabolism (*frhB*, *mcrG*, *fwdD*, and *ftr*) are genomically correlated with average daily gains at early growing periods (ADG_1_ and ADG_2_), mostly positively (see Additional file 5: Table S3). One hypothesis to explain these positive r_g_ could rely on the genetic determination for retention time of digesta (RTD) varying among animals. Variations in RTD among cattle could affect average daily gain by altering the abundance and activity of microorganisms in the rumen, the energetic efficiency of their growth, and also the digestibility of the diet [69]. A higher RTD is associated with a greater release of digestion by-products to be directed as nutrients for the host [69], but also with a greater release of substrates for methane production [79]. The objective of this work was exclusively focused on growth rate; however, a selection strategy on the microbiome could be explored to obtain more productive animals that emit less methane. For example, *alr*-MG involved in metabolic pathways that limit the excess of metabolic H_2_ substrate (e.g. reductive acetogenesis), consume H_2_ in alternative pathways other than methane production (e.g. nitrogen fixation), or inhibit methanogenic organisms (e.g. branched-chain amino acids) proposed in our previous microbiome-driven breeding strategy to reduce methane emissions [60], could be incorporated in the current selection criterion.

In our efforts to uncover the taxonomy of the RUG that are major carriers of the 533 *alr*-MG, in our population [40], we found *uncultured Lachnospiraceae bacterium, Ruminococcaceae bacterium uncultured Ruminococcus sp.* and *uncultured Prevotellaceae* bacterium. Interestingly, Li et al*.* [24] found five SNPs in the host genome being associated with unclassified *Lachnospiraceae* and *Ruminococcus* genera. Some of these SNPs were also associated with feed conversion efficiency in beef cattle [24], and were located within the host gene *RAPH1,* which is involved in the ability of the rumen epithelia to absorb nutrients in cattle [70]. In addition, the abundance of the genus *Ruminoccocus* was used as a selection criterion to reduce growth rate in the selection experiment in pigs, while *Prevotella* was used to increase this trait [78].

Other microbial functions with discordant r_g_ directions with average daily gain during the finishing period are critical for the synthesis of cell-membrane components including peptidoglycan genes (*spoVD* and *ftsI*), lipopolysaccharide genes (*lpxK, lpxH*, *lptA*, *lptB*, *lptC*, and *ABC-2.LPSE.P*) and their O-antigen component of lipopolysaccharides (*wecE*, *wecB*, *pseB* and *pseF*), which are essential in the colonization of Gram-negative bacteria [80] (e.g. members of the *Lachnospiraceae* family). Bacterial lipopolysaccharides are microbiota-derived endotoxins [81, 82] that contribute to host metabolic endotoxemia [83, 84] by boosting intestinal permeability [83, 85–87] and triggering proinflammatory responses in various tissues, including muscle [88], when they bind to CD_14_, TLR_4_ [89] muscle receptors. Lipopolysaccharides have been reported to impair insulin action on glucose metabolism in muscle [88], and in extreme cases, elevated circulating levels have been associated with age-related reductions in muscle mass in humans [22]. Our study suggests that the signalling action by lipopolysaccharides, some of which mediated by receptors regulated by host genes (e.g. *TLR*_*4*_) [90–92], may influence muscle development in growing cattle. Individuals with greater butyrate production in their microbiomes are reported to counteract this effect by reinforcing tight function assembly, which could prevent translocation of endotoxins and circulating inflammation [93, 94]. This matches with our finding of the *alsD* gene involved in the butyrate metabolism and its positive r_g_ with average daily gains at early stages (r_g_ was 0.15 (P_0_ = 0.75) with ADG_1_ and 0.29 (P_0_ = 0.91) with ADG_2_).

It is expected that animals grow faster when they have a greater supply of amino acids from microbial proteins synthesised in the rumen, although it has been suggested that this association is not so straightforward. Several *alr*-MG involved in the biosynthesis of phenylalanine, tyrosine and tryptophan (*aroF*, *trpC* and *trpCF*) and in the metabolism of cysteine and methionine (*mmuM*, *E4.4.1.11* and *yrrT*) or arginine (*alaA* and *E3.5.3.1*) had non-zero r_g_ with some growth rates traits and showed an almost entirely inconsistent pattern.

We also found evidence of a genomic association (P_0_ < 0.85) between *alr-*MG involved in sulphur metabolism and ADG_2_, ADG_3_ or ADG_4_. These included anaerobic sulphite reductase genes (*asrB* and *asrC*), *NFU1* and *dmsB* that are involved in the synthesis of iron-sulphur clusters, and sulfhydrogenase genes (*hydB*, *hydG*) or *thiS*, *tusA*, *moeB* and *MOCS2B* that are involved in the sulphur relay system. In the rumen, the sulphur cycle plays a key role in the synthesis of amino acids (e.g., as a component for their iron/sulphur clusters). Most of the sulphur-containing amino acids (cysteine and methionine) are contained in the microbial proteins available for host uptake, since just a few ruminal bacteria require them for their own growth [95]. The incorporation of sulphur into amino acids occurs in its sulphite form after assimilatory sulphate reduction [96], but sulphate can alternatively be used as an electron acceptor to generate H_2_S in dissimilatory sulphate reduction [96]. Sulphite reductases are key enzymes regulating assimilatory and dissimilatory sulphate reductions, which may be related to the synthesis of these aminoacids and explain the genomic association between these *alr-*MG and longitudinal growth rate.

## Conclusions

This work contributes to filling the current gap in the knowledge of the role of microbial metabolism in the growth pattern of beef cattle. Our results indicate that the host genomics influences the functional rumen microbiome, which is an important biological factor contributing to variation in growth rate during the finishing phase of cattle. For example, host genes that influence microbial gene abundances involved in microbial growth and activity, sulphur and amino acid metabolism and transport, or lipopolysaccharides signalling are influenced by the host genomic background that underlies longitudinal average daily gains. Genomic selection for these specific microbiome functions are proposed as a strategy for breeding for an enhanced growth rate at different stages of the finishing period, although the genomic parameters to be introduced in the models need to be estimated with high accuracy. The potential benefits of using microbiome information to estimate the genetic merit of growth traits can be maximized when combined with weight records that are relatively cost-effective to collect. Our findings contribute to understanding the role of microbial metabolism in the growth trajectory of cattle and provide insights for introducing microbiome information into future genomic breeding programs to improve responses to selection for production traits by permanently shaping the rumen microbiome into a more efficient ecosystem.

### Supplementary Information

**Additional file 1**: **Table S1.** Description of the animals used in the study. Experimental design displaying the number of animals within each breed, diet and experiment.

**Additional file 2: Table S2.** Heritabilities of the microbiome. Genomic parameter estimates for the 3631 alr-transformed microbial gene abundances and genetic parameters estimates for 100 randomly selected alr-transformed abundances of microbial genes when using different degrees of freedom in the chi-squared priors for the random effect variances.

**Additional file 3:** **Figure S1.** Robustness analysis to test the sensitivity of the heritability estimates from different prior information. (**a**). Four different scaled-inverse Chi-squared distributions (degrees of freedom (df0) equal to 1, 5, 10 and 20) used as priors for genomic variances in the Bayesian estimation of variance components of 100 randomly selected alr-transformed microbial abundances. As an example, the graph shows the distributions used for alr-transformed microbial abundance of K03623. Scale parameter (S0) is equal to 0.48, calculated as S0 = var (y) *(df0 +1+ number of traits) * R^2^, var (y) being the phenotypic variance of the trait (0.148), and R^2^ being 0.5. (**b**). Heritability estimates (means of the marginal posterior distributions) of the 100 alr-transformed microbial gene abundances when using different prior information.

**Additional file 4:** **Figure S2.** Test of the significance of heritabilities by a permutation test. Distribution of 1000 heritability (h^2^) estimates when the data are permuted compared to the h^2^ estimates with real phenotypes (in red) for 20 randomly selected alr-transformed microbial gene abundances.

**Additional file 5: Table S3.** Results from the bivariate genomic models between growth traits and microbiome traits. Genetic parameters estimates of alr-transformed abundances of microbial genes and each average daily gain when using different degrees of freedom in the inverse Wishart distributions for the genomic effects.

**Additional file 6: Figure S3.** Robustness analysis to test the sensitivity of our estimates from different prior information. Estimated 583 genomic correlations (means of the marginal posterior distributions) of the alr-transformed microbial gene abundances and any of the longitudinal average daily gains estimated assuming different prior information for genomic effects; this is, an inverse Wishart distribution with different prior degrees of freedom (df): 2, 3 (default), 10 and 20. Scale parameter was var (y) *(df0 +1+ number of traits) * R2, var (y) being the phenotypic 2x2 (co)variance matrix between traits, and R2 being 0.5. Prior distribution for the residual (co)variance was an inverse Wishart distribution with df = 5 and S0 = var (y) *(df0 +1+ number of traits) * R2. Only genomic correlations with a probability of being higher or lower than 0 ≥0.85 when using default priors are displayed (n=583).

**Additional file 7:** **Table S4.** Identification of which rumen uncultured genomes carry the microbial genes of interest. Proteins clustered in KEGG orthologous groups (KO) of interest for the microbiome-driven breeding strategy to increase average daily gains at different stages identified in rumen uncultured genomes (RUG).

**Additional file 8: Table S5.** Description of the two subsets of alr-MG selected to be included in genomic evaluations. Genomic parameters of the 32 alr-transformed microbial gene abundances (alr-MG) considered in the microbiome-driven breeding strategy that aims at increasing growth rate at all stages.

## Data Availability

Metagenomic sequence reads for all rumen samples are available in the European Nucleotide Archive (ENA) under accession projects PRJEB31266, PRJEB21624, PRJEB10338 and PRJEB70912.
