# Supplementary figures and images for: Including microbiome information in a multi-trait genomic evaluation: a case study on longitudinal growth performance in beef cattle
Source: Genet Sel Evol. 2024 Mar 15;56:19. doi: 10.1186/s12711-024-00887-6 (PMC10943865; doi:10.1186/s12711-024-00887-6)

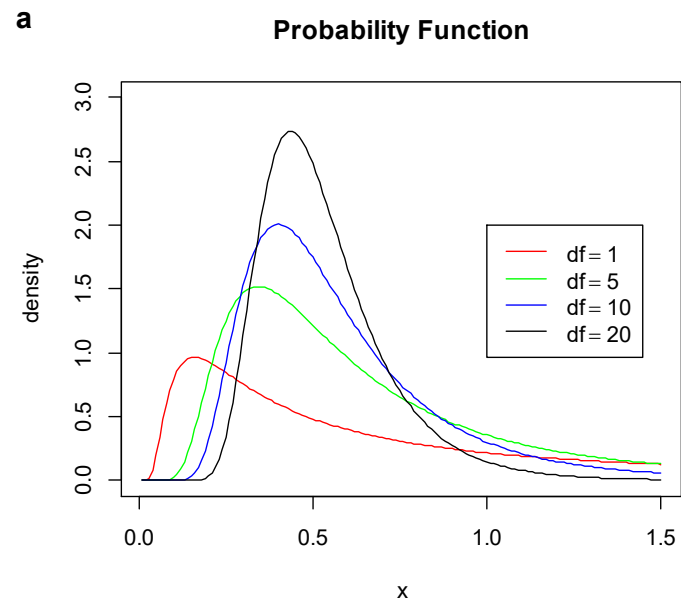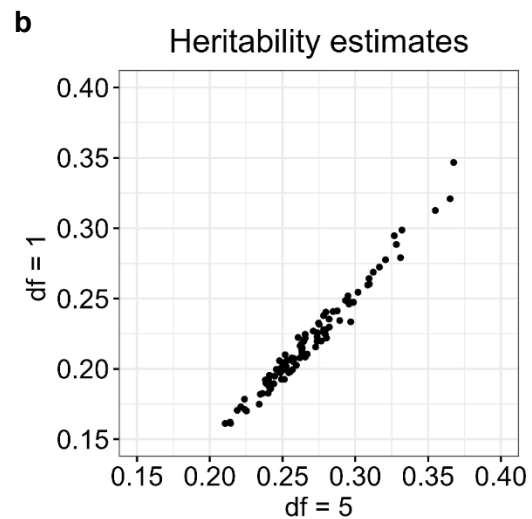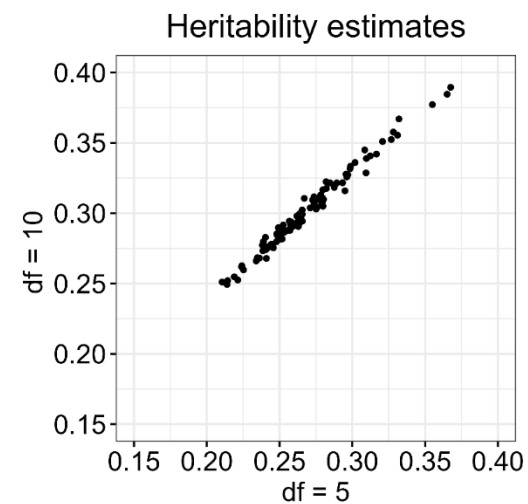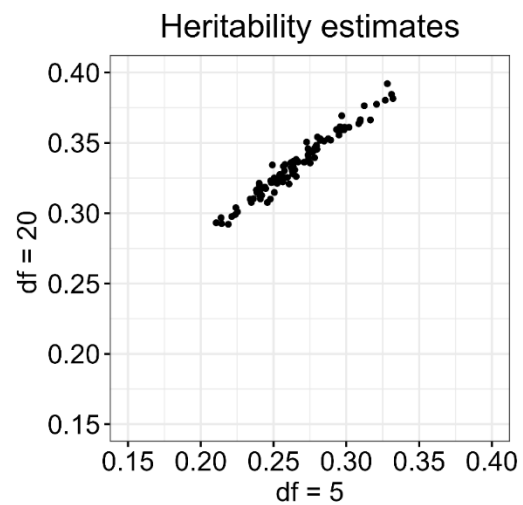

Supplement: Supplementary file 3 — Additional file 3: Figure S1. Robustness analysis to test the sensitivity of the heritability estimates from different prior information. (a). Four different scaled-inverse Chi-squared distributions (degrees of freedom (df0) equal to 1, 5, 10 and 20) used as priors for genomic variances in the Bayesian estimation of variance components of 100 randomly selected alr-transformed microbial abundances. As an example, the graph shows the distributions used for alr-transformed microbial abundance of K03623. Scale parameter (S0) is equal to 0.48, calculated as S0 = var (y) *(df0 +1+ number of traits) * R2, var (y) being the phenotypic variance of the trait (0.148), and R2 being 0.5. (b). Heritability estimates (means of the marginal posterior distributions) of the 100 alr-transformed microbial gene abundances when using different prior information. [file 12711_2024_887_MOESM3_ESM.pdf]

**K07130.K01783**

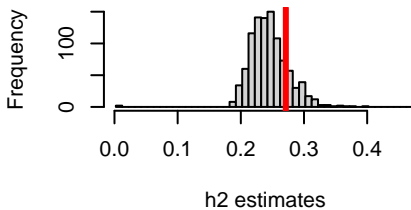

**K07133.K01783**

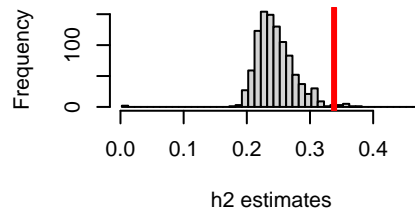

**K07137.K01783**

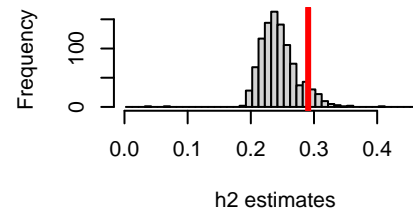

**K05895.K01783**

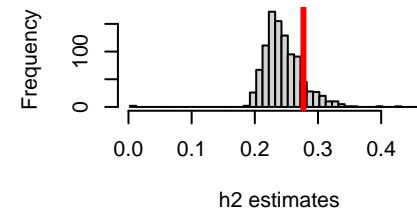

**K03704.K01783**

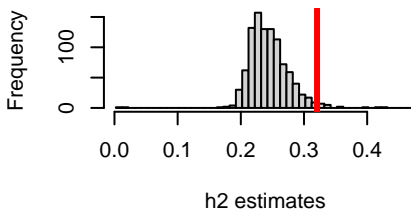

**K03705.K01783**

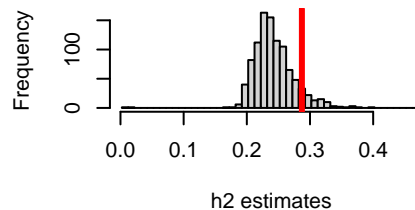

**K09787.K01783**

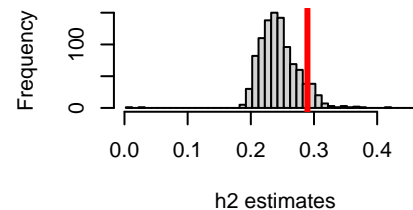

**K03708.K01783**

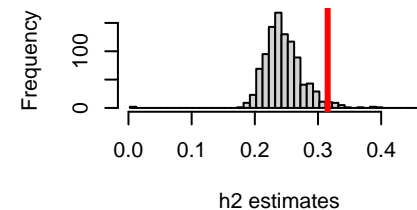

**K16329.K01783**

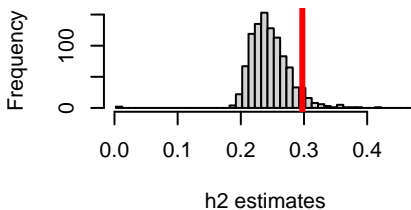

**K07149.K01783**

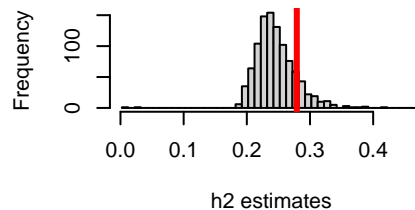

**K03269.K01783**

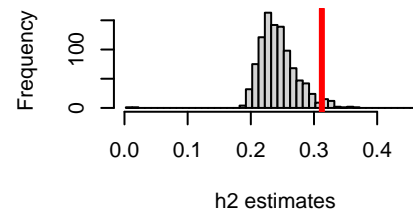

**K07150.K01783**

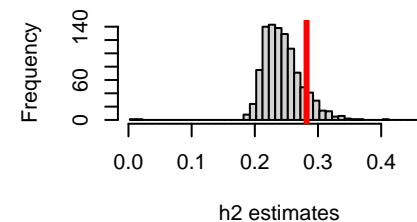

**K07154.K01783**

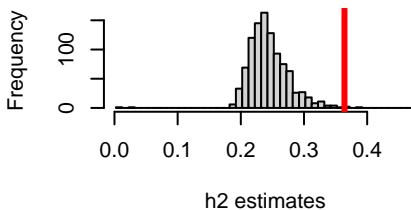

**K03724.K01783**

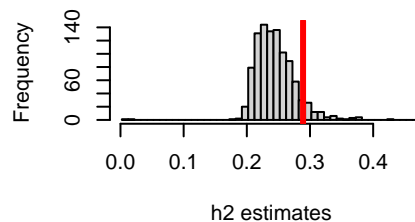

**K08483.K01783**

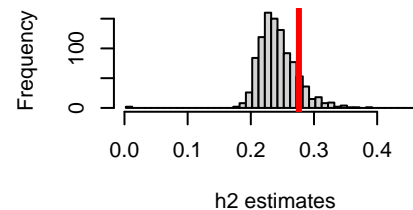

**K07164.K01783**

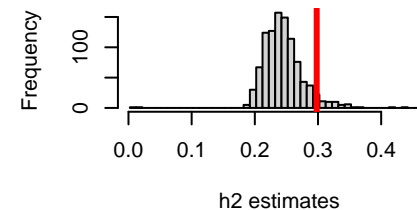

Supplement: Supplementary file 4 — Additional file 4: Figure S2. Test of the significance of heritabilities by a permutation test. Distribution of 1000 heritability (h2) estimates when the data are permuted compared to the h2 estimates with real phenotypes (in red) for 20 randomly selected alr-transformed microbial gene abundances. [file 12711_2024_887_MOESM4_ESM.pdf]

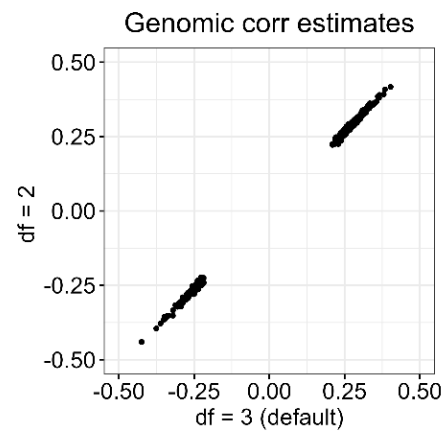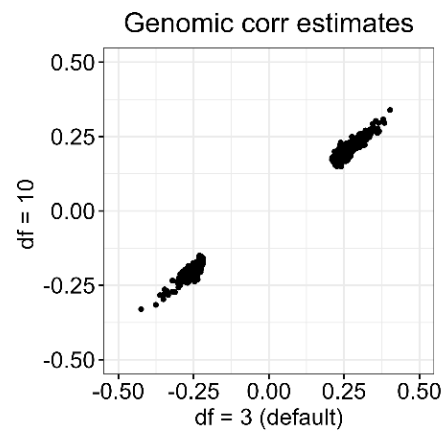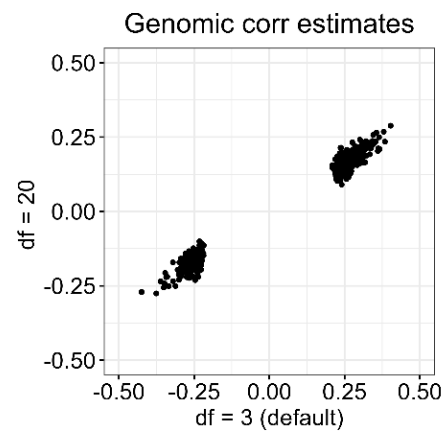

Supplement: Supplementary file 6 — Additional file 6: Figure S3. Robustness analysis to test the sensitivity of our estimates from different prior information. Estimated 583 genomic correlations (means of the marginal posterior distributions) of the alr-transformed microbial gene abundances and any of the longitudinal average daily gains estimated assuming different prior information for genomic effects; this is, an inverse Wishart distribution with different prior degrees of freedom (df): 2, 3 (default), 10 and 20. Scale parameter was var (y) *(df0 +1+ number of traits) * R2, var (y) being the phenotypic 2x2 (co)variance matrix between traits, and R2 being 0.5. Prior distribution for the residual (co)variance was an inverse Wishart distribution with df = 5 and S0 = var (y) *(df0 +1+ number of traits) * R2. Only genomic correlations with a probability of being higher or lower than 0 ≥0.85 when using default priors are displayed (n=583). [file 12711_2024_887_MOESM6_ESM.pdf]
